# Transient Scrotal Hyperthermia Induces Lipid Droplet Accumulation and Reveals a Different ADFP Expression Pattern between the Testes and Liver in Mice

**DOI:** 10.1371/journal.pone.0045694

**Published:** 2012-10-04

**Authors:** Mingxi Liu, Lin Qi, Yan Zeng, Yang Yang, Ye Bi, Xiaodan Shi, Hui Zhu, Zuomin Zhou, Jiahao Sha

**Affiliations:** State Key Laboratory of Reproductive Medicine, Department of Histology and Embryology, Nanjing Medical University, Nanjing, Jiangsu, China; Clermont Université, France

## Abstract

**Background:**

In most mammals, the testes provide a stable environment for spermatogenesis, which depends on a lower temperature than the core body temperature. It has been reported that mild testicular heating safely and reversibly suppresses spermatogenesis, and is under consideration for its potential application as a male contraceptive. Previously, we focused on the molecular mechanism of germ cell apoptosis and anti-apoptotic factors induced by heat treatment in humans and mice. However, the recovery process remains under investigation.

**Results:**

In this study, we found that lipid droplets in mouse testes are dramatically increased after a brief period of scrotal hyperthermia, and gradually dissipate following temperature normalization. Analysis of the human testis proteome revealed nine proteins associated with lipid droplets. Two of them, ADFP (also known as ADRP and PLIN2) and TIP47 (also known as PLIN3) may participate in acute lipid droplet formation in mammalian testes. We show that Adfp expression is upregulated after scrotal heat treatment in mice. Surprisingly, we find *Adfp* lacking its 5′-UTR is observed in *Adfp* ^Δ1/Δ1^ mouse testes, but is not detectable in liver.

**Conclusions:**

These results reveal testis *Adfp* transcriptional regulation is tissue-specific, and is associated with lipid droplet accumulation induced by heat. The results also indicate that the testes could retain functional proteins through testes-specific transcriptional regulation.

## Introduction

Spermatogenesis in mammals takes place in the testes [1]. The adult testes of most mammalian species, including humans, are located extra-abdominally and function at a temperature that is 2°C to 4°C lower than the core body temperature [2]. Testicular heating, which can be caused by saunas, hot baths or by wearing close-fitting underwear, could inhibit spermatogenesis [3], [4]. It has been reported that mild testicular heating safely and reversibly suppresses spermatogenesis in several mammalian species, including humans [5], [6], mice [7], [8], rats [9], [10], monkeys [11], bulls [12] and sheep [13]. Morphological changes in the testes after transient exposure of the scrota to heat are marked by germ cell loss in humans [14], rats [10], mice [7] and monkeys [11] via stage- and germ cell-specific apoptotic pathways. In recent studies, we examined the suppressive effect of testicular heat treatment on spermatogenesis in humans [14] and mice [15]. We have also studied the molecular mechanism of the effect of heat on spermatogenesis, primarily in germ cells [15], [16].

In the present study, we found an abnormality in the testis and epididymis after a temporary heat stress. This result could not be explained by the effect of heat on germ cells alone. Indeed, morphological abnormalities of the Sertoli cells could also affect spermatogenesis [17]–[19]. In mammalian testes, lipid droplets are mainly located in Leydig cells and Sertoli cells, although some lipid droplets have been reported in germ cells, specifically in the residual body of elongated spermatids [20], [21]. It is known that lipid droplets accumulate in severe testicular injury, such as cryptorchidism [22], irradiation [23] and vitamin E deficiency [24].

Recently, transient lipid droplet accumulation following mild testicular hyperthermia has been reported in rats [25], [26]. Lipid droplets contain a core of neutral lipid surrounded by a phospholipid monolayer and are coated by specific proteins [27]. In the present study, we used a mouse model for scrotal transient heat stress as previously described [16], [28], [29]. We determined that testicular lipid droplets dramatically increased following a brief period of scrotal hyperthermia, and that they gradually regressed to normal levels after a week of recovery. Following analysis of the human testis proteome (data not published), we focused on adipose differentiation-related protein (ADFP, also known as ADRP and PLIN2) and tail-interacting protein of 47 kDa (TIP47, also known as PLIN3), which are members of the perilipin (PLIN) family of lipid droplet associated proteins. ADFP binding to lipid droplets is generally limited to adipocytes, steroidogenic cells and other tissues which accumulate lipids [27]. It has been demonstrated that absence of ADFP expression reduced lipid droplet formation and can protect against fatty liver [30]. TIP47 expression occurs in the same cell types as ADFP [27] and can functionally compensate for it [31], [32].

Both ADFP and TIP47 may participate in acute lipid droplet formation in mammalian testes. In this study, we prove that Adfp expression is up-regulated in the testes after heat treatment in mice. Surprisingly, we find testicular Adfp expression was not affected in *Adfp* ^Δ1/Δ1^ gene trapping mouse, but hepatic Adfp expression is ablated. These results reveal that *Adfp* transcription is specifically regulated in the testes, and associated with lipid droplet accumulation induced by heat treatment.

## Results

### Heat shock of 42°C for 30 min reversibly suppresses spermatogenesis

**Figure 1:**
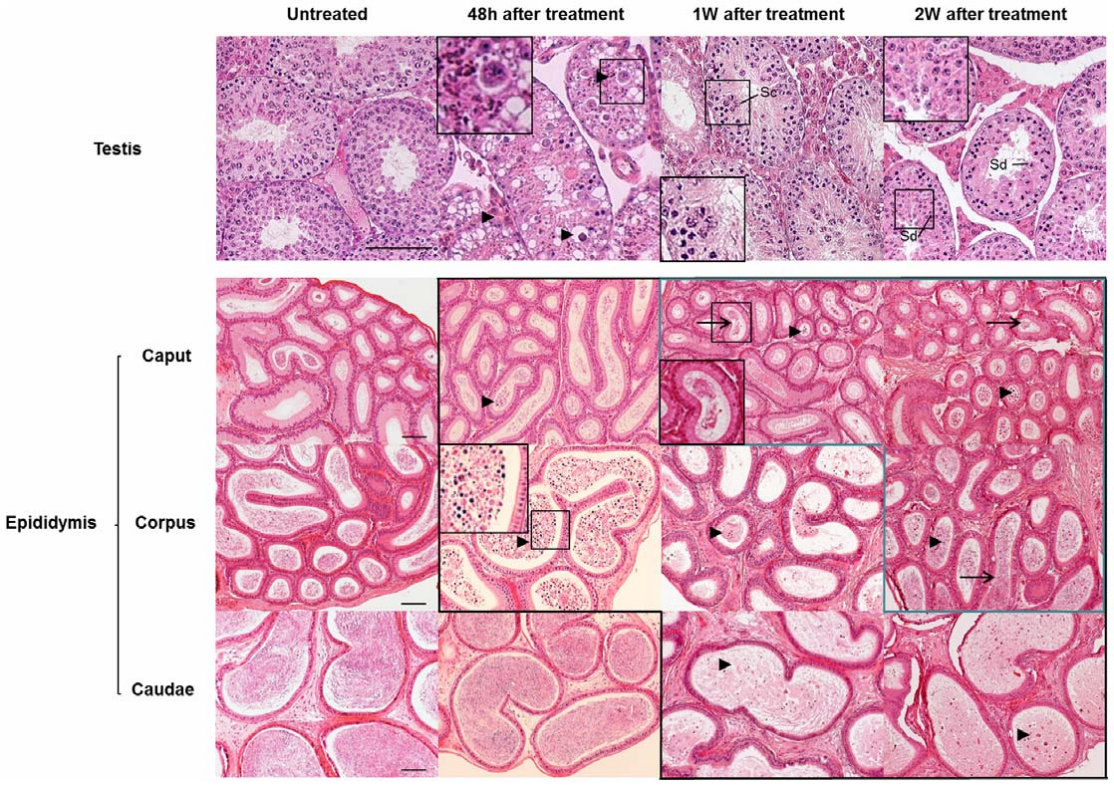
Morphology of normal and heat treated testes was revealed by hematoxylin and eosin staining. Microscopic examination reveals that degenerated and apoptotic germ cells (black triangles) are visible in testes harvested 48 hours after a 42°C heat shock treatment, and at different time points in the epididymis (picture with black frame). Sperm (black arrows) are detectable in the recovered epididymis (picture with blue frame). Scale bar = 50 µm, Sc: spermatocyte, Sd: spermatid. A high magnification image is shown in the black rectangle.

Testicular and epididymal tissues were harvested from four groups of mice: 12 hours, 48 hours, 1 week, 2 weeks and 6 weeks after a 42°C heat shock for 30 min, and untreated controls. Histological examination with hematoxylin and eosin (Figure 1) revealed acidophilic, coagulated germ cells in the seminiferous epithelium 48 hours after heat treatment. Most spermatocytes were affected, and the corpus and caput of the epididymis appeared full of abnormal cells, which were probably cast off from spermatogenic epithelium. After a 1-week recovery period, new spermatocytes appear in injured tubules which did not contain spermatid cells, and abnormal cells were still visible in the epididymis. Two weeks after heat treatment, most spermatogenic tubules recovered with the appearance of spermatid cells, and sperm was seen in the corpus and caput of the epididymis. After 6 weeks, the morphology of all seminiferous epithelium had completely recovered (Figure S1). We have previously reported that heat can cause germ cells to undergo apoptosis and agglutination [14]. In the present study, the TUNEL assay revealed that germ cells that agglutinate undergo apoptosis in the mouse (Figure S2). We further analyzed the testosterone and LH levels in two groups that were untreated and 48 hours after treatment. Serum testosterone and LH levels were significantly increased after treatment (Figure S3), which may have been caused by the loss of germ cells in the seminiferous tubule. This finding indicates that steroidogenesis is activated after heat injury.

### Lipid droplets are dramatically increased after brief scrotal hyperthermia and regress gradually

**Figure 2:**
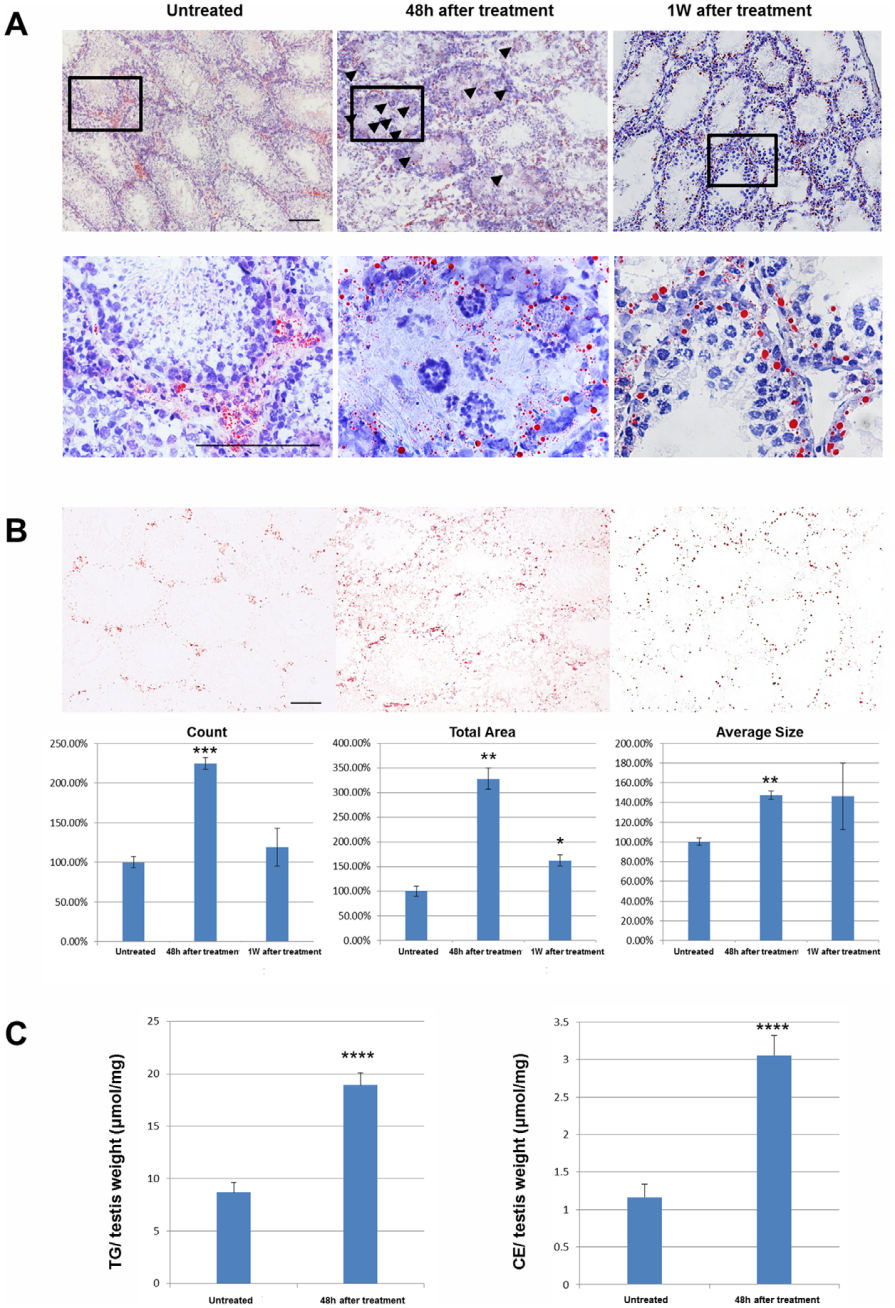
Morphological examination of lipid droplets was performed by oil red O staining (red) and counterstaining using hematoxylin (blue). A high magnification image of regions in the black rectangle is shown in the lower panels. Black triangles point to degenerated and apoptotic germ cells (A). Oil red O staining was quantified by counting number, total area, and average size of staining from discontinuous slides, using three samples per group (B). Bars represent the mean ± SEM, Scale bar = 50 µm. (C) The lipid composition (cholesterol ester [CE] and triglyceride [TG]) in the droplets of testes from the untreated and the 48 hours after treatment groups. (*P<0.01, **P<0.001, ***P<0.0001, ****P<0.00001 compared to the untreated control).

Lipid droplets were visualized by oil red O (ORO) histological staining. In normal mouse testes, lipid droplets in the seminiferous epithelium were generally small in size and number, with some variation was evident between the different spermatogenic stages. Forty-eight hours after heat treatment, there was a dramatic accumulation of lipid droplets. However, after 1 week of recovery, the number of lipid droplets had returned to the normal level, although a few large lipid droplets remained at the base of the seminiferous epithelium (Figure 2A). These observations were confirmed by the measurement of the ORO stained lipid droplet number, total area and average size using ImageJ software (Figure 2B). We further analyzed the lipid composition of the testes in untreated mice and those 48 hours after treatment. The cholesterol ester (TE) and triglyceride (TAG) levels were significantly increased after treatment (Figure 2C).

### Identification of lipid particle associated proteins in testis by bioinformatic analysis of the human testis proteome

To explore the molecular mechanism associated with lipid droplet accumulation, the identity of proteins associated with lipid particles was considered. We analyzed the cellular component of the human testis proteome which contained 7346 proteins (data not published) using Gene Ontology from the David bioinformatics database (http://david.abcc.ncifcrf.gov/) [33]. A list of human testicular proteins associated with lipid particles, and their mouse homologs are provided in Table 1. Two candidates from the PLIN family of lipid droplet associated proteins, ADFP and TIP47, were further researched.

**Table 1:** List of proteins associated with testicular lipid particles.

Human EntrezGene ID	Mouse EntrezGene ID	Gene Name	Gene Description
51099	67469	ABHD5(CGI-58)	abhydrolase domain containing 5
857	12389	CAV1	caveolin 1, caveolae protein, 22 kDa
858	12390	CAV2	caveolin 2
11104	23924	KATNA1	katanin p60 (ATPase containing) subunit A 1
123	11520	PLIN2 (ADFP)	perilipin 2,adipose differentiation-related protein,Adipophilin
729359	57435	PLIN4 (S3–12)	perilipin 4, Plasma membrane associated protein, S3–12
10226	66905	PLIN3 (TIP47)	perilipin 3,Cargo selection protein TIP47
27339	28000	PRPF19	PRP19/PSO4 pre-mRNA processing factor 19 homolog (S. cerevisiae)
10280	18391	SIGMAR1	sigma non-opioid intracellular receptor 1
90627	243362	STARD13	StAR-related lipid transfer (START) domain containing 13

### Adfp and Tip47 are located on the surface of lipid droplets in mouse testis after heat treatment

**Figure 3:**
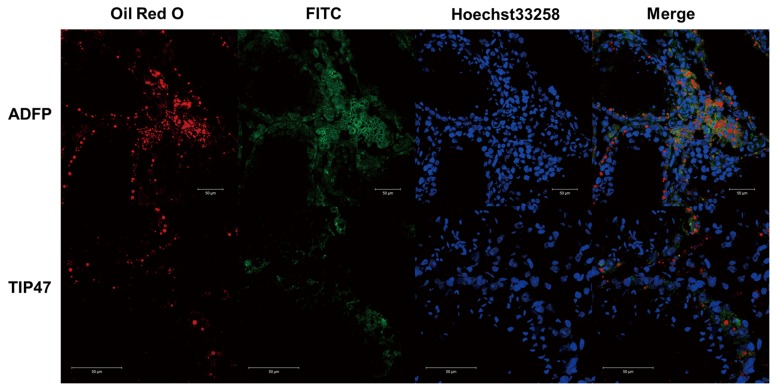
Immunofluorescence of testicular ADFP and TIP47 in the group 1 week after treatment. ADFP and TIP47 are labeled by FITC (green), lipid droplets are labeled by Oil red O (red), and nuclei are labeled by Hoechst 33258 (blue) in frozen sections of fresh testicular tissue. Scale bar = 50 µm.

After 1 week of recovery, Adfp surrounded most lipid droplets, especially the large lipid droplets at the base of the seminiferous epithelium. Tip47 was not located at the surface of large lipid droplets, but was closely associated with a few small lipid droplets and dispersed through the cytoplasm in a variety of cell types in the seminiferous epithelium (Figure 3). In comparison with untreated controls (data not shown), Adfp was located on the surface of lipid droplets and Tip47 had more generalized expression in Leydig cells, Sertoli cells and germ cells in testes from different time points after heat treatment.

### Adfp expression was upregulated by heat treatment

**Figure 4:**
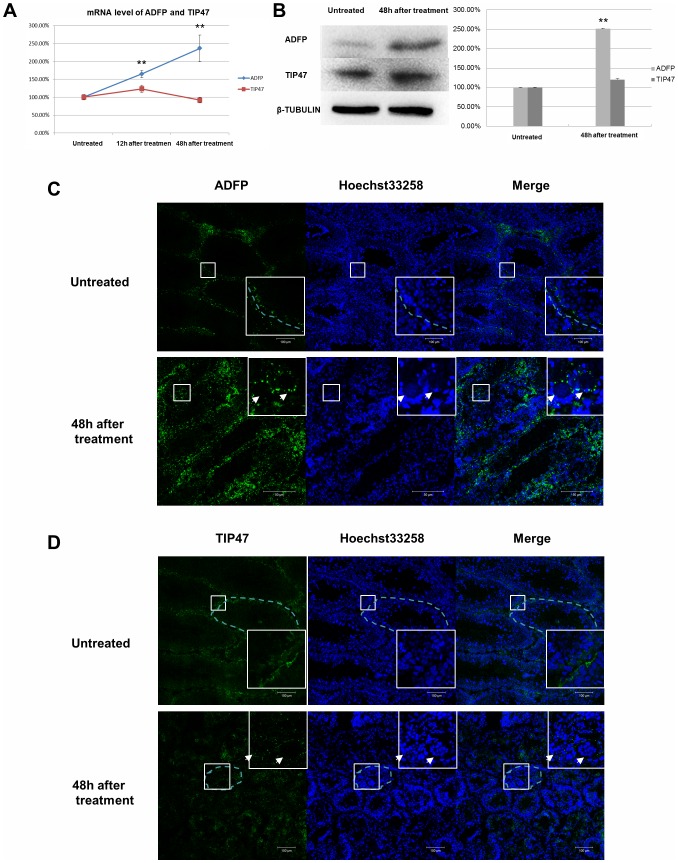
The mRNA of *Adfp* and *Tip47* level was compared using quantitative RT-PCR. *Adfp* expression increased 12 hours and 48 hours after heat treatment (A). The protein expression level of Adfp and Tip47 was measured by Western blotting. Adfp is increased in the mouse testes 48 hours after heat treatment (B). Indirect immunofluorescence of Adfp (C) and Tip47 (D) reveals increasing intensity of Adfp 48 hours after heat treatment, especially in the seminiferous tubules associated with large numbers of degenerated and apoptotic germ cells (white arrow). **P<0.001, Scale bar = 100 µm.

The level of *Adfp* mRNA was significantly increased after heat treatment, as determined by qRT-PCR (Figure 4A). Western blot analysis revealed that the quantity of Adfp protein was also upregulated (Figure 4B). Tip47 expression in the testes was not notably changed (Figure 4A, B). Indirect immunofluorescence revealed that Adfp staining was increased in seminiferous tubules, especially in the impaired tubules (Figure 4C). Interestingly, we observed that the Tip47 staining appeared as small round signals that surrounded acidophilic, coagulated germ cells, which was similar to the pattern of Adfp expression (Figure 4D).

### Testicular Adfp expression in the *Adfp^Δ1/Δ1^* mouse

**Figure 5:**
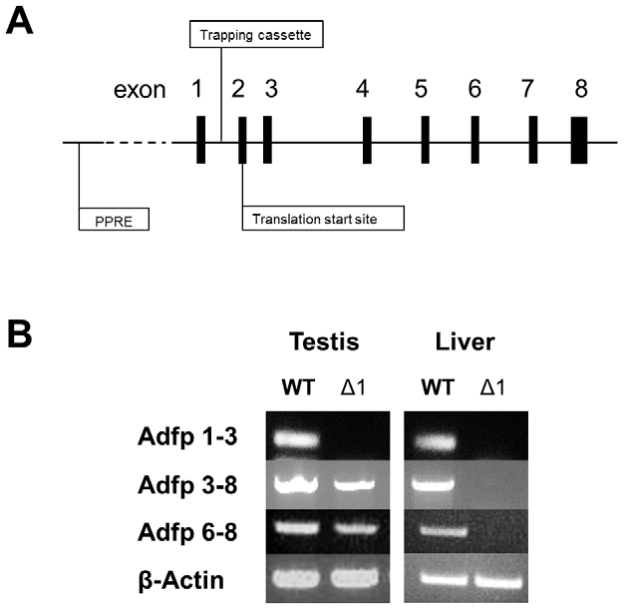
The trapping cassette of *Adfp* ^Δ1/Δ1^ mice is inserted in the intron between exons 1 and 2, before the *Adfp* translation start site. The peroxisome proliferator-activated receptor responsive element (PPRE) is located ∼2,000 bp upstream of the transcription start site (A). RT-PCR products are presented using primers targeted to exons 1–3, exons 3–8, and exons 6–8 of the *Adfp* transcripts of testes and liver from *Adfp* ^Δ1/Δ1^ mice and wild type mice. β-actin was amplified as the control gene (B).

Plin2^Gt(OST170322)Lex^ mice were generated by insertion of a trapping cassette in the intron between exons 1 and 2 (exon 2 contains the translation start site) of the *Adfp* gene (Figure 5A). Homozygote Plin2^Gt(OST170322)Lex^ mice will subsequently be referred to as *Adfp* ^Δ1/Δ1^ mice. RT-PCR data using primers targeted to exons 3–8 and exons 6–8 of murine *Adfp* shows that *Adfp* transcripts were present in the testes, but not the livers of *Adfp* ^Δ1/Δ1^ mice. Additionally, data using primers targeted to exons 1–3 of mouse *Adfp* shows an absence of 5′-UTR mRNA from both the testes and liver from *Adfp* ^Δ1/Δ1^ mice (Figure 5B).

## Discussion

### Lipid droplets accumulation increasing in testis may supply the material for spermatogenic recovery

Lipid transport from Sertoli cells to germ cells is important for spermatogenesis [34], [35]. Recently, it has been demonstrated in vitro that lipid β-oxidation is a major pathway for the production of ATP, and that phagocytosis of apoptotic spermatogenic cells increases ATP production and lipid droplet accumulation in the Sertoli cells of mouse testes [36]. In cryptorchid rat testes, which could be considered as hyperthermic, the amount and concentration of triglycerides and cholesterol esters increased several fold [37]. In the current study, we found that transient scrotal hyperthermia induced lipid droplet accumulation in murine testes. It is known that distinct testicular injuries can induce lipid droplet accumulation in addition to hyperthermia, such as irradiation [23] and vitamin E deficiency [24]. We hypothesized that, after testicular injury, Sertoli cells could phagocytose and degrade apoptotic cells, storing the excess lipid component in lipid droplets. During recovery from injury, lipid droplets are then utilized to provide energy for metabolism and biosynthesis of germ cells.

### Testicular proteins located in lipid droplets

In our list of testicular proteins associated with lipid droplets, Caveolin1 and Caveolin 2, which are transmembrane scaffolding proteins from the Caveolin protein family, may play a transport function of caveolae [38]. Another protein, ABHD5 (also known as CGI-58) channels fatty acids released from the hydrolysis of stored triacylglycerols into phospholipids [39]. Notably, ADFP, TIP47and S3–12 (also known as PLIN4), which are all members of the PLIN family of lipid droplet associated proteins, could bind the membrane of lipid droplets and may have a role in lipid droplet formation. The newly identified member of the PLIN family, PLIN4, which is structurally different with typical PLIN proteins [27] is not further discussed in this research.

### Modified testicular ADFP expression is an independent mechanism of lipid regulation from liver

An evolutionarily conserved peroxisome proliferator-activated receptor (PPAR)-response element is located ∼2,000 bp upstream of the transcription start site in mouse and human *ADFP*, and *ADFP* is known to be transcriptionally regulated by PPAR-alpha (PPARα) in mouse liver [40]. In this study, we detected an *Adfp* transcript in the testes of *Adfp* ^Δ1/Δ1^ mice. Another *Adfp* mutant mouse model, which lacks exons 2 and 3, is a complete knock-out, with no *Adfp* transcripts detectable in the testes [41]. Furthermore, there was no *Adfp* transcript detectable in the liver of these mice, or in the liver of *Adfp* ^Δ1/Δ1^ mice. These results suggest that the transcriptional regulatory region for *Adfp* in testicular tissue is near exon 2, which interestingly contains the translation start site for this gene. The transcriptional control of testicular *Adfp* expression may not be as sensitive as hepatic *Adfp* to the multiple ligands for PPARα from blood. It is still not clear why 5′UTR is missing in *Adfp* transcripts in the testis of *Adfp* ^Δ1/Δ1^ mice. Our results indicate that the mechanism of lipid regulation in the testis is independent from that in the liver. This phenomenon might contribute to the maintainance of local lipid homeostasis in the testis.

## Materials and Methods

### Animals

This mouse study was approved by the ethics committees of Nanjing Medical University and was in accordance with the national and international guidelines. Plin2^Gt(OST170322)Lex^ mice were bred at the animal center of Nanjing Medical University (Nanjing, China), and were originally transferred from the Mutant Mouse Regional Resource Center (MMRRC). The strain information can be found at the following website: http://www.mmrrc.org/strains/11683/011683.html. Mice were housed under specific pathogen-free conditions with unlimited access to food and water. Genotyping was performed using the provided MMRRC protocol (http://www.mmrrc.org/strains/11683/di_protocol.pdf).

### Induction of transient heat stress

Male, wild-type mice, aged 8 to 9 wk were subjected to a single heat stress of 42°C for 30 min. Each animal was anesthetized, and the lower third of the body, including the hind legs, tail, and scrotum, were submerged in a 42°C water bath. Control animals were anesthetized and left at room temperature. After 30 min, each animal was dried, and returned to its cage.

### RNA extraction, RT-PCR and quantitative RT-PCR

Total RNA was extracted from adult testes from each control and heated group using the RNeasy Plus Micro Kit with on-column DNase digestion (Qiagen Ltd., Crawley, West Sussex, UK). Random primed cDNA was prepared using the PrimeScript™ RT Master Mix (TaKaRa Bio Inc., Otsu, Japan). Primer sequences are presented in the Supporting Information (Table S1). The various cDNAs were PCR-amplified with specific primers in 20 µl of GoTaq Green Master Mix (Promega Corporation, CA, USA). The amplification conditions consisted of initial denaturation at 94°C for 5 min, followed by 30 cycles at 94°C for 30 sec, 55°C for 30 sec, and 72°C for 30 sec. The final extension was carried out at 72°C for 10 min. The PCR products were analyzed by 1.5% w/v agarose gel electrophoresis, using mouse β-actin as the control gene. Quantitative RT-PCR was performed using the ABI PRISM 7300 sequence detection system (Applied Biosystems) using the following thermal cycling conditions: 30 sec at 95°C; followed by 40 cycles of 5 sec at 95°C, 31 sec at 55°C, and 30 sec at 72°C. Beta-actin was amplified in parallel as a loading control.

### Western blot analysis

Samples that contained 100 µg of protein from adult mouse testes were electrophoresed and transferred to a nitrocellulose membrane (Amersham Biosciences, Uppsala, Sweden). The membranes were blocked and then incubated overnight with primary antibodies against Adfp (Abcam, Cambridge, UK) and Tip47 (Abcam) at a dilution of 1∶200. Membranes were then washed and incubated for 1 h with a horseradish peroxidase conjugated anti-rabbit IgG secondary antibody at a dilution of 1∶2,000 (Beijing Zhong-Shan Biotechnology Co.). Proteins were detected using an ECL kit and AlphaImager (Amersham).

### Oil red O staining

ORO (0.5%) was dissolved in 70% ethanol and filtered to remove undissolved particles. For lipid staining, cells were fixed in 4% paraformaldehyde in PBS for 15 min, washed with PBS, then incubated with ORO for 10 min, and finally washed in PBS to remove unincorporated staining solution. Staining was assessed by bright-field microscopy and quantified by ImageJ 1.43u software (National Institutes of Health, Bethesda, Maryland, USA) after appropriate thresholding. There were four samples in each group (untreated, 48 hours after treatment and 1 week after treatment). Three discontinuous slides of each mouse from each group were used for the analysis.

### Lipid profile measurements

The testes from four mice in each group (untreated and 48 hours after treatment) were collected and the lipid profiles were determined with an enzymatic analysis using a tissue triglyceride assay kit and a cholesterol ester assay kit (Applygen, Beijing, China).

### Serum testosterone and LH assessment

Serum samples from four mice in each group (untreated and 48 hours after treatment) were collected and determined with a RD Mouse Testosterone(T) Elisa kit and a RD Mouse Luteinizing Hormone Elisa kit (Nanjing Jiancheng Bioengineering Institute, Nanjing, China).

### TUNEL assay

DNA fragmentation as an index of apoptosis was determined by the TUNEL assay (Roche Diagnostics, Indianapolis, IN) according to the manufacturer's specifications using paraffin sections of the mouse testis.

### Indirect immunofluorescence and combined oil red O and immunofluorescence staining

Frozen sections of fresh testicular tissue were fixed with 4% paraformaldehyde in PBS for 40 min and permeabilized with 0.2% Triton X-100 in PBS for 20 min at 37°C. After blocking in PBS containing calf serum for 2 h, samples were incubated overnight with a 1∶200 dilution of antibodies against Adfp (Abcam) and Tip47 (Abcam) at 4°C. Samples were then incubated with an anti-rabbit IgG secondary antibody labeled with FITC (Beijing Zhong-Shan Biotechnology Co.) at a 1∶100 dilution for 1 h at room temperature. Combined ORO and immunofluorescence staining was performed as previously described, with a slight modification [42]. Briefly, after ORO staining was performed, the above immunofluoresence protocol was followed, continuing from the blocking step. ORO can be excited at wavelengths between 540 and 580 nm, similar to Texas red. Slides were viewed with an LSM710 confocal microscope (Zeiss, Germany) driven by ZEN2009 software (Zeiss).

### Statistical analysis

All experiments were repeated at least three times. The differences between treatment and control groups were analyzed using one-way ANOVA. Values of P<0.05were considered to be statistically significant. All data represent the mean plus or minus the standard error of the mean.

## Supporting Information

Table S1 The List of Primers.
(XLS)

Figure S1 **The morphology of seminiferous epithelium after treated 6 weeks.**
(TIF)

Figure S2 **The TUNEL assay of seminiferous epithelium after treated 48 hours.**
(TIF)

Figure S3 **The serum testosterone and LH levels in two groups (untreated and 48 hours after treatment).**
(TIF)
