# Supplementary figures and images for: Transient Scrotal Hyperthermia Induces Lipid Droplet Accumulation and Reveals a Different ADFP Expression Pattern between the Testes and Liver in Mice
Source: PLoS One. 2012 Oct 4;7(10):e45694. doi: 10.1371/journal.pone.0045694 (PMC3464254; doi:10.1371/journal.pone.0045694)

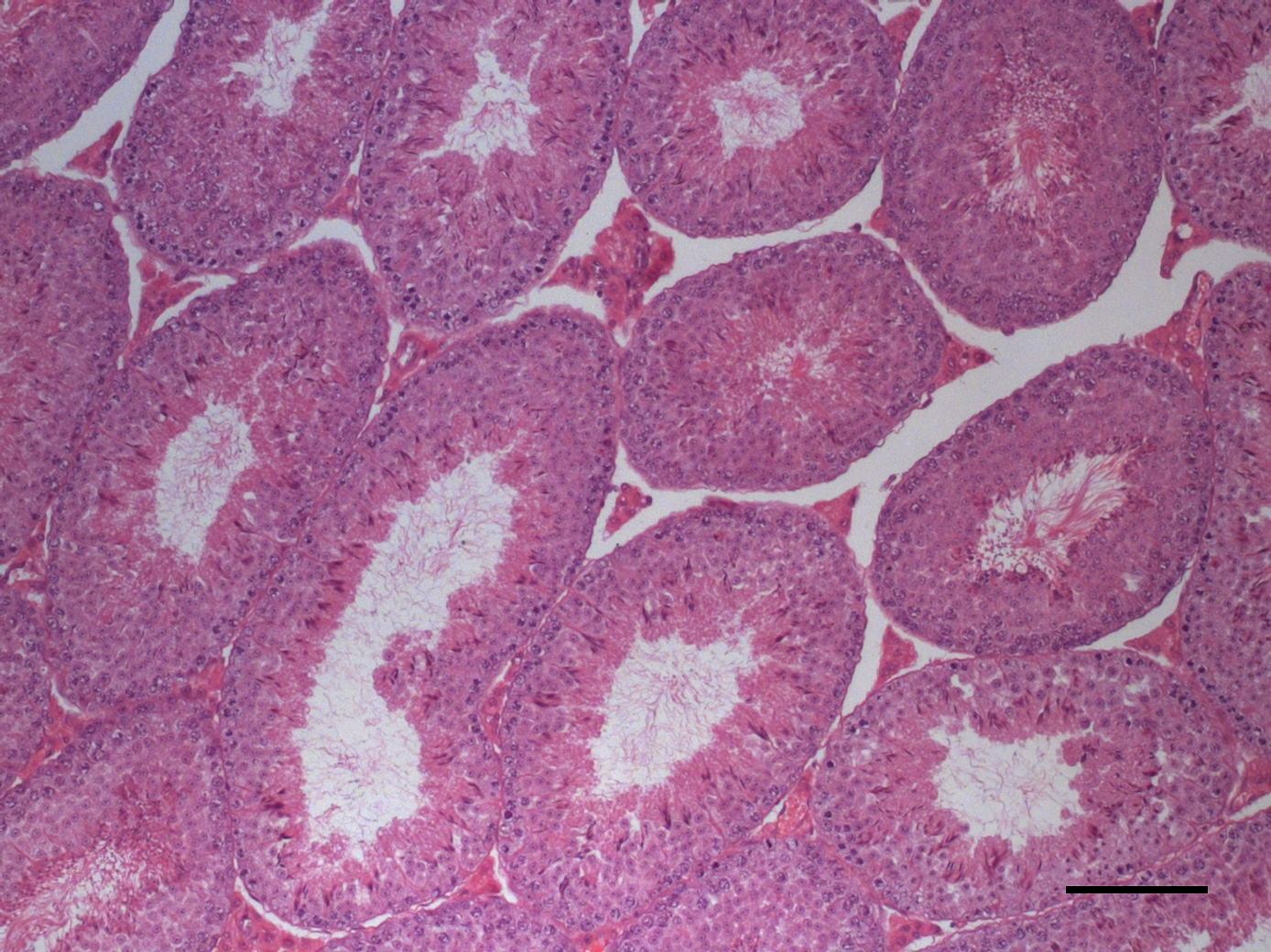

Supplement: Figure S1 — The morphology of seminiferous epithelium after treated 6 weeks. (TIF) [file pone.0045694.s002.tif]

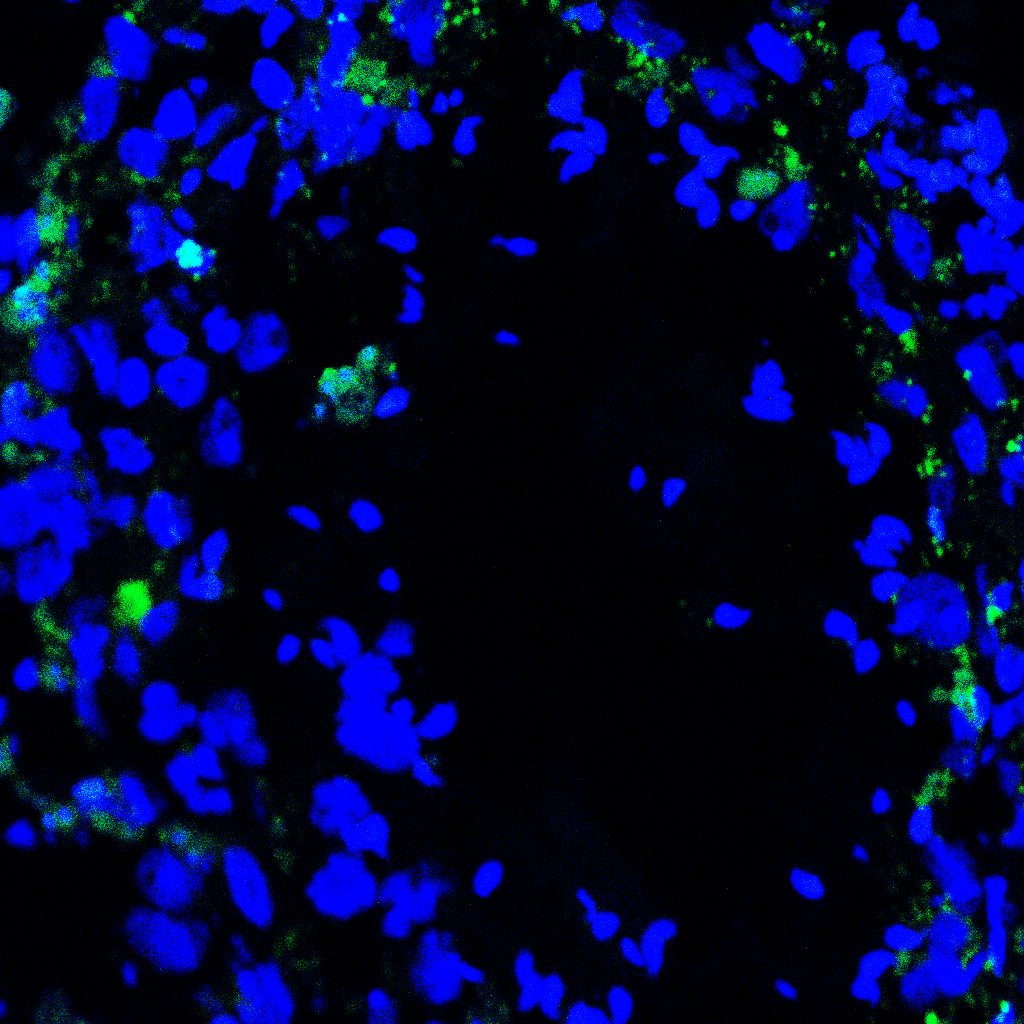

Supplement: Figure S2 — The TUNEL assay of seminiferous epithelium after treated 48 hours. (TIF) [file pone.0045694.s003.tif]

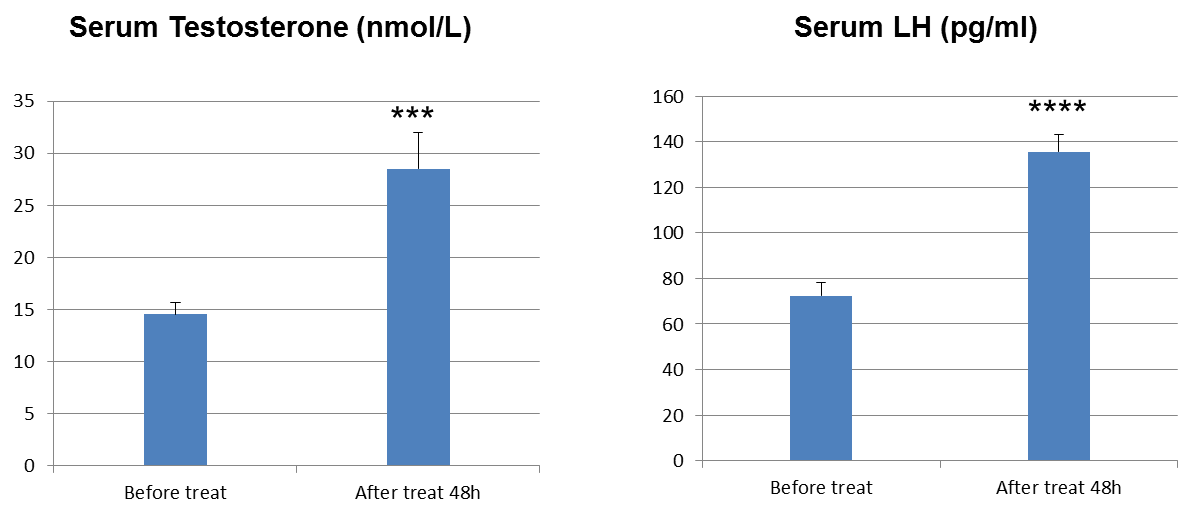

Supplement: Figure S3 — The serum testosterone and LH levels in two groups (untreated and 48 hours after treatment). (TIF) [file pone.0045694.s004.tif]
